# Cyclin-dependent kinase 4/6 inhibitors and interstitial lung disease in the FDA adverse event reporting system: a pharmacovigilance assessment

**DOI:** 10.1007/s10549-020-06001-w

**Published:** 2020-11-05

**Authors:** Emanuel Raschi, Michele Fusaroli, Andrea Ardizzoni, Elisabetta Poluzzi, Fabrizio De Ponti

**Affiliations:** 1 Pharmacology Unit, Department of Medical and Surgical Sciences, Alma Mater Studiorum - University of Bologna, Bologna, Italy; 2 Medical Oncology Unit, Department of Experimental, Diagnostic and Specialty Medicine, Policlinico S. Orsola-Malpighi, Alma Mater Studiorum - University of Bologna, Bologna, Italy

**Keywords:** Cyclin-dependent kinase (CDK) 4/6 inhibitors, Abemaciclib, Interstitial lung disease, Pharmacovigilance, FAERS, Signal

## Abstract

**Purpose:**

We assessed pulmonary toxicity of cyclin-dependent kinase (CDK)4/6 inhibitors by analyzing the publicly available FDA Adverse Event Reporting System (FAERS).

**Methods:**

Reports of interstitial lung disease (ILD) were characterized in terms of demographic information, including daily dose, latency, concomitant drugs known to be associated with ILD, and causality assessment (adapted WHO system). Disproportionality analyses were carried out by calculating reporting odds ratios (RORs) with 95% confidence interval (CI), accounting for major confounders, including notoriety and competition biases.

**Results:**

ILD reports (*N* = 161) represented 2.1% and 0.3% of all reports for abemaciclib and palbocilcib/ribociclib, respectively, with negligible proportion of concomitant pneumotoxic drugs. Increased reporting was found for CDK4/6 inhibitors when compared to other drugs (ROR = 1.50; 95%CI = 1.28–1.74), and abemaciclib vs other anticancer agents (4.70; 3.62–5.98). Sensitivity analyses confirmed a strong and consistent disproportionality for abemaciclib. Higher-than-expected reporting emerged for palbociclib (1.38; 1.07–1.77) and ribociclib (2.39; 1.34–3.92) only when removing Japan reports. ILD occurred at recommended daily doses, with median latency ranging from 50 (abemaciclib) to 253 (ribociclib) days. Causality was *highly probable* in 55% of abemaciclib cases, *probable* in 68% of palbociclib cases.

**Conclusions:**

Increased reporting of ILD with CDK4/6 inhibitors calls for further comparative population-based studies to characterize and quantify the actual risk, taking into account drug- and patient-related risk factors. These findings strengthen the role of (a) timely pharmacovigilance to detect post-marketing signals through FAERS and other real-world data, (b) clinicians to assess early, on a case-by-case basis, the potential responsibility of CDK4/6 inhibitors when diagnosing a lung injury.

## Introduction

The cyclin-dependent kinase (CDK)4/6 inhibitors – palbociclib, abemaciclib, and ribociclib – are now approved both in the United States and Europe for women with hormone receptor-positive, human epidermal growth factor receptor 2-negative advanced breast cancer, based on positive results of several large pivotal phase III randomized controlled trials [1–3].

From a safety standpoint, CDK4/6 inhibitors are well-tolerated agents, with similar safety profile, although some differences exist in the pattern and frequency of toxicities, which might influence the choice of a given medication. The most common side effect for palbociclib and ribociclib is neutropenia, whereas gastrointestinal toxicity is associated especially with abemaciclib (showing less selectivity for CDK4, which plays a critical role in hematopoietic stem cell differentiation) [4]. Among rare adverse events, higher frequency of QT prolongation emerged for ribociclib, whereas transaminases increase was recorded with ribociclib and abemaciclib resulting in regulatory warnings [5].

Pulmonary toxicity was reported for several anticancer drugs, and over 1300 medications, procedures, or substances are considered to cause respiratory impairment (www.pneumotox.com), although the pulmonary liability is often recognized after marketing approval. The term “interstitial lung diseases” (ILDs) poses a challenging clinical diagnosis as it refers to heterogeneous disorders with remarkably different clinical pathophysiology, histories, and prognoses, such as idiopathic pulmonary fibrosis, sarcoidosis, connective tissue disease associated with ILDs, and hypersensitivity pneumonitis. Drug-induced ILD incidence rates vary between 4.1 and 12.4 cases/million/year, with anticancer agents, anti-rheumatic drugs, amiodarone, and antibiotics being the most common causes [6].

Although data on pulmonary toxicity with CDK4/6 inhibitors are scant, recent case reports have described the potential occurrence of pneumonitis, including fatal cases [7]. Moreover, in the Food and Drug Administration (FDA) label for abemaciclib, it is reported that there was 1 death due to pneumonitis in the MONARCH 3 trial, and 2 deaths due to pneumonitis in the MONARCH 2 trial. In the MONALEESA-2 trial, there were two deaths secondary to acute respiratory failure in patients receiving ribociclib plus letrozole. In May 2019, the Japanese Ministry of Health released a warning as 14 patients in Japan developed pulmonary toxicity likely due to abemaciclib exposure (nearly 2000 exposed), and three of them died. Recently, on September 13th, 2019, the FDA issued a warning about rare but severe lung inflammation with CDK4/6 inhibitors, based on cases of ILD and pneumonitis identified in the manufacturers’ completed and ongoing clinical trials and the post-marketing safety databases. Although rare (1–3% of patients had ILD/pneumonitis of any grade), there were serious cases (less than 1% had fatal outcome), and some patients had at least one risk factor (https://www.fda.gov/drugs/drug-safety-and-availability/fda-warns-about-rare-severe-lung-inflammation-ibrance-kisqali-and-verzenio-breast-cancer).

Post-marketing monitoring is therefore crucial to timely characterize ILD and to target preventive strategies for diagnosis and management [8]. In this context, international spontaneous reporting systems, through collection of millions of worldwide reports, represent a primary source of data for safety assessment of recently marketed drugs receiving fast track designation and priority review, which deserve rigorous post-marketing monitoring [9, 10]. In particular, the FDA Adverse Event Reporting System (FAERS) is the largest publicly available pharmacovigilance database particularly suitable to detect rare adverse events, which may escape detection and/or reporting from randomized controlled trials.

On these grounds, the aim of this real-world post-marketing study is to comprehensively characterize spontaneous reports of ILD with CDK4/6 inhibitors submitted to FAERS and investigate whether pulmonary toxicity actually occurs with all CDK4/6 inhibitors.

## Methods

### Study design and data source

The study was conceived as an observational, retrospective pharmacovigilance analysis of the FAERS database, which has gained considerable interest in the medical community [10]. As of March 31st, 2020, FAERS collected more than 20 million raw reports, covering virtually worldwide population (relevant catchment area includes also serious reports from EU and other non-US Countries) and can be analyzed both through interactive web-based tool (the so-called FAERS public dashboard) or by downloading raw data for customized search, as performed in the present study.

To evaluate if, and to what extent, pulmonary toxicity has been reported with CDK4/6 inhibitors more frequently than for other drugs, we performed a disproportionality analysis, a consolidated approach in pharmacovigilance, to identify potential drug–event associations, by comparing the proportion of selected adverse events (AEs) reported for a single drug or drug class (e.g., CDK4/6 inhibitors) with the proportion of the same AEs for a control group of drugs. Conventionally, the denominator in these analyses is the total number of reports recorded in FAERS. If the proportion of AEs is greater in patients exposed to a specific drug (cases) than in patients not exposed to this drug (non-cases), an association can be hypothesized between the specific drug and the event [11].

Through this so-called *case/non-case* approach, which can be viewed as a case–control analysis, the reporting odds ratio (ROR) with relevant 95% confidence interval (95%CI) was calculated using the 2 × 2 contingency table and deemed statistically significant when the lower limit of the 95%CI of the ROR exceeds 1 with at least 3 cases reported, as recommended [12, 13]. The performance of disproportionality studies through ROR is noteworthy (i.e., the capacity to discriminate true from false-positive drug–event associations), especially for AEs with low/rare background incidence and a likely drug-attributable component such as ILD [14].

### Case and exposure definition

Pulmonary toxicity encompasses a variegate spectrum of lung diseases, with different clinical phenotypes, as well as varying histopathological patterns, even with the same offending drug, including pneumonitis (auto-immune, eosinophilic, and hypersensitivity pattern), pulmonary fibrosis, sarcoidosis, and pleural effusion [6].

In this study, pulmonary toxicity was specifically assessed by focusing on ILD, i.e., using only the term ILD, codified through the Medical Dictionary for Regulatory Activity (MedDRA) terminology. This choice was guided by pharmacological and clinical aspects: among the various manifestations of pulmonary toxicity, ILD carries the strongest drug-induced component, whereas other phenotypes of lung involvement might be the expression of the underlying complications of the disease (metastatic pleural effusion), relevant therapeutic approaches (e.g., radiation pneumonitis), or underlying infective complications (bronchiolitis, pneumonia). This choice was also undertaken to reduce the likelihood of false-positive results.

Different groups of exposure of interest were considered, including CDK4/6 inhibitors as a pharmacological class as well as individual agents (abemaciclib, ribociclib, palbociclib). Bleomycin served as a positive control, considering its well-known association with ILD. In this study, exposure assessment considered drugs recorded as suspect (‘primary suspect,’ ‘secondary suspect’) and concomitant. Sensitivity analyses were performed by restricting disproportionality to suspect cases.

### Disproportionality approach

Pharmacovigilance in oncology poses several challenges, such as multiple therapeutic regimens, poly-pharmacotherapy, comorbidities, and drug–drug and drug–disease interactions [15, 16]. Moreover, the unique benefit–risk consideration may result in a higher threshold for recognizing and reporting AEs.

Therefore, a list of pre-specified analyses was planned to assess consistency of findings and minimize major biases. First, primary disproportionality analyses were carried out (up to March 2020) by comparing CDK4/6 inhibitors with: (a) all other drugs reported in the FAERS database (a traditional pharmacovigilance approach); (b) other oncological drugs (using AEs recorded for at least one anticancer agent), in order to provide a clinical perspective; this so-called analysis by therapeutic area can be viewed as intra-class analysis and also reduce the so-called indications bias (in case the disease can be a risk factor for AE occurrence) by selecting a real-world subpopulation that presumably shares at least a set of common risk factors [17].

Second, the following sensitivity analyses were performed: (a) to account for potential notoriety bias by relevant FDA warning, only reports up to September 2019, i.e., before the FDA warning, were analyzed [18]; (b) to test the existence of event-related competition biases (i.e., a masking effect by widely known and highly reported toxicities) [19], we removed high-frequency events recorded with CDK4/6 inhibitors, namely diarrhea and neutropenia, using relevant Standardized MedDRA queries; (c) to test the impact of concomitant medications, we removed drugs known to be strongly associated with ILD [6], which may act as confounding factor and may also mask the identification of ILD with CDK4/6 inhibitors (the so-called drug-related competition bias) [19]; (d) to explore the role of Ethnicity and considering the high reporting of ILD with anticancer drugs in Japan [20–22], we restricted the analyses among non-Japanese reports; (e) to test the primary role of CDK4/6 inhibitors, analyses were restricted to reports where the agents were recorded as suspect (i.e., primary/secondary suspect); (f) to account for marketing approval, a restricted dataset (2015–March 2020) was selected considering that palbociclib, the first-in class drug, was approved in February 2015. Analyses were performed through the open-source R software (version 4.0.2; 2020-06-22).

### Causality assessment

Cases of ILD by CDK4/6 inhibitors were described in terms of demographic characteristics, including age, reporter country (US, Europe, Asia), reporter type (e.g., clinician vs consumer), fatality (i.e., death reported as the outcome), seriousness (focusing on events resulting in hospitalization), latency (i.e., time to onset of the ILD, expressed in days with interquartile range – IQR -, calculated as the difference between the start of therapy and the date the event occurred), daily dose intake, dechallenge (clinical improvement after the offending agent is suspended), and rechallenge (occurrence of a similar reaction after re-administration, usually unintentional).

Individual cases were assessed for causality (categorized as certain, highly probable, probable, possible, unlikely) according to an adaptation of the standardized WHO-UMC system (https://www.who.int/medicines/areas/quality_safety/safety_efficacy/WHOcausality_assessment.pdf). Assessment criteria are based on plausibility of time relationship to drug intake, lack of concomitant drugs potentially explaining the event, positive dechallenge/rechallenge. Specifically, drugs with strong evidence of ILD were selected from the systematic review by Skeoch et al. [6], including anticancer and anti-rheumatic agents. Highly probable cases were those with plausible time to onset, alternate drugs ruled out, and positive dechallenge. Certain cases were defined in case positive rechallenge was also recorded.

Causality assessment was also carried out on the entire body of evidence, by using adapted Bradford Hill Criteria, widely used in epidemiology, which can be applied to pharmacovigilance; they accounted for biological plausibility, strength, consistency, specificity, coherence, and analogy [23, 24].

## Results

### Descriptive analyses

A total of 25,503 cases of ILD were found in FAERS, of which 161 (0.6%) with CDK4/6 inhibitors, peaking in 2009 (88 cases); a 2-fold increase was noted in the ratio between ILD cases and other events for CDK4/6 inhibitors, as compared to 2018. ILD represented 2.1% of total reports recorded for abemaciclib, as compared to 0.3% for palbociclib and ribociclib (Table 1).

**Table 1:** Demographic data of interstitial lung disease (ILD). Data are expressed as counts, with relevant percentage in parentheses. In square brackets, corresponding percentage of non-ILD events is presented.

	Abemaciclib	Palbociclib	Ribociclib	CDK4/6 inhibitors†	All other drugs	Other anticancer drugs
***Total cases of ILD***	*60*	*93*	*14*	*161*	*25,503*	*17,800*
**Age distribution**						
Adult	11 (27) [52]	20 (28) [46]	2 (29) [55]	33 (28) [47]	7208 (36) [59]	5479 (39) [63]
18–29	0 (0) [1]	0 (0) [1]	0 (0) [2]	0 (0) [1]	324 (5) [14]	211 (4) [11]
30–50	4 (36) [27]	0 (0) [26]	1 (50) [37]	5 (15) [27]	1859 (26) [38]	1373 (25) [38]
51–64	7 (64) [72]	20 (100) [73]	1 (50) [60]	28 (85) [72]	5025 (70) [48]	3895 (71) [51]
Elderly	30 (73) [48]	52 (72) [54]	5 (71) [44]	84 (72) [53]	12,523 (62) [35]	8409 (59) [32]
65–75	16 (53) [65]	36 (69) [64]	2 (40) [67]	52 (62) [64]	7463 (60) [59]	5494 (65) [66]
76–85	11 (37) [27]	14 (27) [30]	3 (60) [29]	27 (32) [30]	4291 (34) [32]	2604 (31) [28]
>85	3 (10) [7]	2 (4) [6]	0 (0) [4]	5 (6) [6]	769 (6) [9]	311 (4) [5]
Other/Missing	19	21	7	44	5772	3912
**Type of reporter**						
Physician	21 (35) [13]	53 (58) [17]	9 (64) [35]	80 (50) [19]	12,326 (51) [25]	9129 (54) [29]
Consumer	31 (52) [44]	22 (24) [36]	4 (29) [29]	55 (35) [36]	3451 (14) [49]	2427 (14) [42]
Pharmacist	5 (8) [18]	6 (7) [20]	0 [6]	10 (6) [19]	1784 (7) [7]	937 (6) [7]
Other/Missing	3	12	1	16	7942	5307
**Reporter Country**						
Asia/Japan	49 (82) [6]	39 (42) [5]	2 (14) [8]	84 (52) [5]	9109 (53) [6]	7020 (56) [8]
North America	8 (13) [88]	25 (27) [84]	7 (50) [44]	40 (25) [80]	3552 (21) [75]	2519 (20) [73]
Europe	3 (5) [5]	29 (31) [8]	4 (29) [37]	36 (22) [10]	4317 (25) [16]	2823 (23) [16]
Other/Missing	0	0	1	1	8525	5438
**Outcome#**						
Hospitalization	39 (65) [20]	42 (45) [16]	8 (57) [29]	87 (54) [18]	13,618 (53) [25]	9369 (53) [26]
Death	18 (30) [7]	24 (26) [11]	5 (36) [14]	46 (29) [11]	7417 (29) [9]	5797 (33) [10]
Life-threatening	5 (8) [2]	8 (9) [1]	2 (14) [5]	15 (9) [1]	3992 (12) [3]	2138 (12) [3]
Other	27 (45) [20]	68 (73) [32]	7 (50) [51]	99 (61) [33]	14,239 (56) [35]	10,253 (58) [37]
Missing	6 (10) [57]	6 (6) [50]	0 (0) [22]	10 (6) [47]	139 (1) [40]	86 (0) [38]
**Time to onset, days** (IQR; N with available data)	50 (21–71; 35)	85 (19–143; 41)	253 (77–336; 6)	63 (21–136; 82)	71 (21–263; 11,443)	57 (17–154; 8232)
Concomitant ILD drugs*						
Amiodarone	/	4 (4.2)	/	4 (2.5)	1235 (4.8)	208 (1.2)
Gemcitabine	/	2 (2.1)	/	2 (1.2)	672 (2.6)	672 (3.8)
Cetuximab	/	/	1 (7.1)	1 (0.6)	269 (1.1)	269 (1.5)
Everolimus	/	4 (4.2)	/	4 (2.5)	598 (2.3)	598 (3.4)

/ Not reported, *IQR* interquartile range

*Based on the systematic review by Skeoch et al. [6]: amiodarone, methotrexate, leflunomide, cyclophosphamide, bleomycin, gemcitabine, gefitinib, erlotinib, infliximab, etanercept, rituximab, panitumumab, cetuximab, sirolimus, temsirolimus, everolimus, nitrofurantoin, daptomycin

^#^ Multiple outcomes can be reported for the same report

^†^ The sum of the number of ILD cases for CDK4/6 inhibitors as a drug class is lower than the total number of ILD cases for individual CDK4/6 inhibitors because, in a few reports, more than one agent was recorded

Some specific demographic features emerged for ILD, as compared to other events. The majority of cases originated from Asia/Japan (52%), especially for abemaciclib (82%), with the exception of ribociclib, for which North America was the reporter Country in 50% of cases. Most of ILD reports occurred in subjects aged >65 years (72%) and were submitted by physicians (64% for ribociclib), except for abemaciclib (consumers were recorded in 52%). ILD was reported to occur at therapeutic doses (250, 125, and 600 mg daily for abemaciclib, palbociclib, and ribociclib, respectively).

Hospitalization and death were recorded in 54% (65% for abemaciclib) and 29% (36% for ribociclib) of cases, respectively. Median latency (IQR) was 50 (21–71), 85 (19–143), and 253 (77–336) days for abemaciclib, palbociclib, and ribociclib, respectively. The calculated time to onset for bleomycin was 92 (52.5–136.5) days. Only in a negligible proportion of cases were concomitant drugs known to be associated with ILD recorded, namely amiodarone and everolimus (4.2% of palbociclib ILD cases). Causality assessment was *highly probably* in 35% of ILD cases (55% for abemaciclib), *probable* in 61% (68% for palbociclib).

### Disproportionality analyses

Increased reporting of ILD emerged for CDK4/6 inhibitors as a class, when compared to all other drugs recorded in the FAERS database (ROR = 1.50; 95%CI = 1.28–1.74). When the analysis was performed within anticancer agents, only abemaciclib was found with higher-than-expected reporting (4.70; 3.62–5.98), at similar extent to bleomycin (7.19; 6.22–8.27) (Table 2).

**Table 2:** Disproportionality approach: primary analyses

Comparator	CDK4/6 inhibitors†	Abemaciclib	Palbociclib	Ribociclib	Bleomycin
	[*N* = 44,990]	[*N* = 2938]	[*N* = 37,478]	[*N* = 4719]	[*N* = 6438]
	N, ROR (95%CI)	N, ROR (95%CI)	N, ROR (95%CI)	N, ROR (95%CI)	N, ROR (95%CI)
All drugs	161, 1.50 (1.28–1.74)*	60, 8.66 (6.59–11.06)*	93, 1.05 (0.85–1.27)	14, 1.28 (0.74–2.04)	199, 13.20 (11.38–15.16)*
Anticancer drugs	161, 0.81 (0.69–0.94)	60, 4.70 (3.62–5.98)*	93, 0.57 (0.46–0.69)	14, 0.69 (0.40–1.11)	199, 7.19 (6.22–8.27)*

*Statistically significant disproportionality, i.e., lower limit of the 95% confidence interval > 1 (see text for details). ROR: Reporting Odds Ratio; CI: confidence Interval

^†^ The sum of the number of ILD cases for CDK4/6 inhibitors as a drug class is lower than the total number of ILD cases for individual CDK4/6 inhibitors because, in a few reports, more than one agent was recorded

In the sensitivity analyses, abemaciclib consistently emerged with solid disproportionality across all approaches, thus indicating that no major confounders are likely to exist. Only in two circumstances, did palbociclib and ribociclib generate statistically significant ROR (i.e., the signal was unmasked), including correction for concomitant drugs known to cause ILD and, unexpectedly, when restricting the analysis to reports outside Japan, thus refusing the hypothesis of a strong ethnicity-related risk factor (Table 3).

**Table 3:** Disproportionality approach: sensitivity analyses and relevant signal consistency

	Restricted to the 2015–2020 period	Restricted to non-Japan reports	Corrected for event-related competition bias	Corrected for drug-related competition bias #	Corrected for notoriety bias	Restricted to suspect reports	Signal consistency
Drug	N, ROR (95%CI)	N, ROR (95%CI)	N, ROR (95%CI)	N, ROR (95%CI)	N, ROR (95%CI)	N, ROR (95%CI)	
CDK4/6 inhibitors†	161, 1.59 (1.35–1.85)*	83, 1.61 (1.30–1.99)*	122, 1.43 (1.19–1.70)*	154, 1.98 (1.69–2.31)*	122, 1.46 (1.22–1.73)*	158, 1.49 (1.27–1.73)*	**STRONG (6/6)**
Abemaciclib	60, 9.23 (7.10–11.83)*	11, 3.44 (1.82–5.81)*	45, 9.93 (7.25–13.12)*	60, 11.92 (9.15–15.25)*	42, 9.75 (7.04–13.02)*	58, 8.49 (6.48–10.88)*	**STRONG (6/6)**
Palbociclib	93, 1.10 (0.90–1.34)	59, 1.38 (1.07–1.77)*	68, 0.94 (0.74–1.18)	87, 1.34 (1.07–1.64)*	72, 1.00 (0.79–1.26)	92, 1.05 (0.84–1.27)	WEAK (2/6)
Ribociclib	14, 1.37 (0.78–2.21)	13, 2.39 (1.34–3.92)*	13, 1.56 (0.88–2.55)	13, 1.70 (0.96–2.77)	13, 1.76 (0.96–2.86)	13, 1.20 (0.68–1.98)	WEAK (1/6)

*Statistically significant disproportionality, i.e., lower limit of the 95% confidence interval > 1 (see text for details). ROR: Reporting Odds Ratio; CI: confidence Interval.

^#^ Based on the systematic review by Skeoch et al. [6]: amiodarone, methotrexate, leflunomide, cyclophosphamide, bleomycin, gemcitabine, gefitinib, erlotinib, infliximab, etanercept, rituximab, panitumumab, cetuximab, sirolimus, temsirolimus, everolimus, nitrofurantoin, daptomycin

^†^ The sum of the number of ILD cases for CDK4/6 inhibitors as a drug class is lower than the total number of ILD cases for individual CDK4/6 inhibitors because, in a few reports, more than one agent was recorded

Globally, the majority of Bradford Hill criteria were fulfilled, as indicated by ROR strength and its consistency throughout the analyses, temporal relationship, and biological plausibility, thus supporting a likely causal association (Table 4).

**Table 4:** Causality assessment of interstitial lung disease (ILD) with CDK4/6 inhibitors based on Bradford Hill criteria

Criterium	Findings	Criterium fulfilled
Strength of the association	This is the first disproportionality analysis comparing reporting of ILD with CDK4/6 inhibitors in primary analysis (versus all other drugs and anticancer agents). Although ROR is not a measure of risk, the strength of the ROR suggests a strong signal (the impact of unmeasurable confounders is likely to be negligible)	√
Consistency	Results of disproportionality approaches were consistent across various sensitivity analyses, especially for abemaciclib	√
Coherence	Case reports have been recently published suggesting a potential association. Some imbalances emerged from clinical trials, although underpowered to actually identify and characterize rare adverse events	√
Specificity	Although ILD encompasses a heterogeneous spectrum of lung toxicities, we applied the most specific definition to reduce the likelihood of false-positive results. Pharmacovigilance data suggest that a drug-specific effect (rather than a class-effect) cannot be excluded	?
Biological gradient	Although the quality of reports is variable due to missing data, there are no clues of actual dose- or duration-response relationships. ILD was reported to occur at therapeutic dosage (i.e., a collateral effect rather than a toxic effect)	?
Temporal relationship	Notwithstanding variable quality of reports due to missing data, ILD does appear to occur early after drug administration, thus suggesting the need for patients’ monitoring, especially in the presence of risk factors (smoking, previous lung disease, Japanese subjects). This latency is in line with data from anticancer drugs (e.g., EGFR inhibitors and bleomycin). Moreover, positive dechallenge in the majority of cases supports reversibility	√
Biological plausibility and experimental support	A recent pre-clinical study in a mice model of bleomycin-induced lung fibrosis found that palbociclib augmented inflammatory cell recruitment (including macrophages and T cells) in the bronchoalveolar lavage fluid. This may represent a plausible mechanistic basis that may increase patients’ susceptibility to ILD occurrence	√
Analogy	The association of ILD with several anticancer drugs (e.g., EGFR inhibitors, bleomycin, checkpoint inhibitors) and bleomycin (used as positive control) is comparable in terms of ROR	√

*ROR* reporting odds ratio (a measure of disproportionality), *EGFR* epidermal growth factor receptor

√ Relevant criterium is fulfilled based on pharmacovigilance data

? Uncertainty in fulfillment of relevant criterium

## Discussion

In the past few years, the therapeutic scenario of hormone receptor-positive, epidermal growth factor receptor 2-negative, advanced/metastatic breast cancer has been profoundly changed by the clinical availability of CDK4/6 inhibitors. These agents differ in terms of tolerability (gastrointestinal, liver, and bone marrow toxicities), with relevant impact on safe prescribing by oncologists [1–5].

To the best of our knowledge, this is the first large-scale post-marketing safety study investigating the reporting of ILD with CDK4/6 inhibitors. This pharmacovigilance assessment, stemming from recent serious case reports of pneumonitis, characterizes the occurrence of ILD through multiple disproportionality approaches combined with a case-by-case evaluation, a complementary approach only rarely carried out in the literature [25]. Although this pulmonary toxicity appears to be rare, oncologists should be aware that ILD does occur with CDK4/6 inhibitors even in patients without apparent drug-related risk factors, namely concomitant drugs known for their ILD risk.

Overall, we found a consistent increased reporting of ILD with CDK4/6 inhibitors as a class, which was consistent across the various sensitivity analyses accounting for major biases and confounders, especially for abemaciclib. ILD is key feature of Japanese population, as indicated by several post-marketing analyses documenting a higher-than-expected reporting of ILD in Japan, especially for anticancer drugs, possibly due to genetic susceptibility, market penetration, and Japan regulatory system [20–22]. Notably, removal of Japan reports did not have substantial impact on disproportionality for all CDK4/6 inhibitors, and a signal was even unmasked for palbociclib and ribociclib, thus suggesting that a drug-related component is likely to exist. The large reporting of ILD in elderly population (as compared to other events) should be also further explored as a potential host-related risk factor.

Although actual incidence and prevalence cannot be derived from spontaneous reporting, the fact that CDK4/6 inhibitors were reported in 161 cases over the past few months (1.2% out of total 13,450 ILD cases in the past 4 years) suggests that the estimated risk is not so rare as stated in the relevant summary of product characteristics (SPC); this is also in line with a recent polled safety analysis of MONARCH 2 and 3 trials, describing ILD as an infrequent toxicity [26]. Remarkably, the proportion of ILD vs other events exponentially increased in 2009, even before the FDA warning; this trend can be potentially interpreted as a consequence of the increasing uptake of CDK4/6 inhibitors worldwide, especially in progressively less selected populations. Therefore, we fully endorse the FDA warning. The relevant sections in the US and EU labels differ and provide imprecise mention of the relevant ILD profile. Palbociclib was the only agent with a warning in both US and EU SPCs, whereas abemaciclib and ribociclib received a warning in the EU and US SPCs, respectively. We believe that the SPCs should be harmonized, also by adding relevant data in the specific section on side effects, where only data from clinical trials are provided.

When looking at the pneumotox.com (last access July 09th, 2020), a standard reference for ILD, the statement “*pneumonitis/ILD, including acute and severe form (may occasion an ARDS picture),”* is reported for palbociclib and abemaciclib, whereas for ribociclib, a general reference to the FDA warning on CDK4/6 inhibitors as a class is mentioned. We found a *highly probable/probable* causality assessment in the majority of cases, thus corroborating a true drug-related association. Moreover, positive dechallenge supports the reversibility of ILD (reported to occur at therapeutic doses); these clinical elements, together with the observed relatively rapid time to onset (median latency of 63 days for CDK4/6 inhibitors as a class, in line with literature data on anticancer drugs) [27], strengthen the importance of early recognition of signs/symptoms suggestive of ILD (e.g., dyspnea), especially at the beginning of treatment, for appropriate management including drug discontinuation. In MONARCH 2 and 3 trials, only rarely were dose reductions and/or discontinuation required, whereas ¼ of patients were treated with steroids and/or antibiotics [26].

Abemaciclib emerged with a consistent strong disproportionality (i.e., very high ROR values, mirroring the association with bleomycin). Although we recognize that comparative safety studies through pharmacovigilance databases are debated [28], there are no expected major distortions to the data that can potentially explain the observed difference in disproportionalities, and the three agents are broadly comparable in terms of therapeutic uses, while differing from scheduling of administration (continuous for abemaciclib rather than 3-week cycles, with exposure increasing less than proportionally with an increasing dose) and pharmacodynamic standpoints. Abemaciclib possesses unique pharmacological properties as compared to palbociclib and ribociclib; in vitro data have shown that it is the most potent inhibitor of CDK4 and 6, with lower potency against CDK1, CDK7, and CDK9 and also showed direct inhibitory activities against other kinases including glycogen synthase kinase 3α/β and calmodulin-dependent protein kinase II α/β/γ. Moreover, abemaciclib targets the dual specificity tyrosine phosphorylation-regulated kinase as well as homeodomain-interacting protein kinase [29–31]. Taken together, these data should prompt mechanistic studies to investigate whether CDK4/6 or other kinases are potentially involved in ILD pathophysiology. Moreover, the potential relationship between physiochemical/pharmacokinetic features (e.g., lung distribution) and ILD occurrence should be explored; of note, abemaciclib possesses higher lipophilicity vs other CDK4/6 inhibitors. These pharmacological differences might also partially explain the observed differences in the latency (50 days for abemaciclib vs 253 days for ribociclib), although the low number of cases does not allow firm conclusions. From a pharmacokinetic perspective, the three agents share some main features, including metabolism mediated by CYP3A4 (with abemaciclib producing active and abundant metabolites), potentially resulting in drug–drug interactions, and biliary clearance as the main elimination pathway [32].

Because of inherent limitations [10–12, 28], including no certainty on causation, incomplete/missing information, and potential codification errors, these findings raise the hypothesis that a drug-specific susceptibility exists for ILD, and ask per se for additional analytical studies, such as population-based investigation, to confirm the signal before any regulatory action other than information or harmonization of SPCs can be envisioned [30]. In particular, this study cannot be used to quantify ILD risk and provide risk ranking mainly because: (a) under-reporting and the lack of data on population exposure do not actually allow calculation of incidence rate; (b) the diagnosis depends on a number of criteria, including radiological assessment and the exclusion of other causes such as prior radiotherapy, which cannot be obtained with absolute certainty. Moreover, contribution of additional drugs with underlying (but unknown) pulmonary toxicity cannot be excluded, as well as residual confounders. Nonetheless, our real-world analysis has important strengths [33, 34], including a large-scale comprehensive pharmacovigilance assessment, encompassing disproportionality approaches (major confounders ruled out, including notoriety bias) and case-by-case evaluation (WHO and Bradford Hill criteria), which consistently support the notion of a real causal association. While the role of ethnicity deserves further investigation, there are no reasons to support the existence of confounding by indication or channeling bias (i.e., preferential prescription towards more severe patients with risk factors for ILD) and, if present, it should apply to all CDK4/6 inhibitors.

The mechanisms of ILD are unknown, and someone may even consider ILD with CDK4/6 inhibitors a paradoxical event since the anti-proliferative mode of action of these agents can be theoretically useful in counteracting lung fibrosis. However, a recent in vivo study in a mice model of bleomycin-induced lung fibrosis found that, after 14 days, palbociclib significantly decreased collagen deposition in the lung after bleomycin treatment but did not ameliorate lung function. Importantly, palbociclib augmented inflammatory cell recruitment (including macrophages and T cells) in the bronchoalveolar lavage fluid [35]. Mechanistically, the elevated levels of inflammatory cell could be a consequence of the palbociclib-induced cell cycle arrest with relevant cellular senescence, a phenomenon called “senescence associated secretory phenotype.” This supports the biological basis of ILD with CDK4/6 inhibitors and relevant inflammatory-mediated mechanism, which is also compatible with the observed early latency.

## Conclusion

In summary, CDK4/6 inhibitors should be added to the evolving list of drug-induced ILD, an emerging and challenging differential diagnosis especially in the current COVID-19 era. The strong signal for abemaciclib, consistent across various approaches, warrants further investigation through population-based studies, to establish actual event rates and identify risk factors that might lead to proper risk management, including the contributing role of age, ethnicity and pharmacological properties.

These findings support the notion that CDK4/6 inhibitors have different safety profile, and ILD should be considered within the spectrum of rare toxicities together with liver injury and QT prolongation. This should be taken into account for safe prescribing in the individual patient, considering the underlying risk factors and therapeutic alternatives.

The relatively recent marketing approval of CDK4/6 inhibitors, together with their promising place in therapy and relevant expected increasing utilization in clinical practice, suggest the importance of monitoring patients at the beginning of therapy for early signs of lung toxicity and strengthen the role of spontaneous reporting systems as a crucial source to monitor in the real-world the occurrence of serious, rare, and unpredictable toxicities. Detailed reporting can provide valuable opportunities to identify safety signals and ultimately protect patients’ safety.

## Data Availability

The datasets analyzed during the current study are available in the following resource available in the public domain: https://fis.fda.gov/extensions/FPD-QDE-FAERS/FPD-QDE-FAERS.html.
